# Obituary

**Published:** 1878-03-20

**Authors:** 


### OBITUARY.

Dr. Edward F. Knott, formerly of Griffin, and who resided for a short time in Atlanta, died, in Hampton, Georgia, on the 24th of February last, at the age of 69 years. Dr. Knott was a teacher of medicine many years ago in Georgia, and acquired considerable eminence in his profession. He was the father of our medical friend and fellow-citizen, Dr. J. J. Knott, of Atlanta, and leaves many warm and devoted friends to mourn his departure.

---

MISFIT CARPETS.—See advertisement of J. A. Bendall, importer and dealer in foreign and domestic carpets. Doctors as well as other people need carpets, and like to know where to get good bargains in this line.

---

LUDDEN & BATES are the leading piano and music men in the South. We have tried one of their instruments and find it in all respects what they represent. See their advertisement.

